# Optical Oxygen Sensing and Clark Electrode: Face-to-Face in a Biosensor Case Study

**DOI:** 10.3390/s22197626

**Published:** 2022-10-08

**Authors:** Pavel V. Melnikov, Anastasia Yu. Alexandrovskaya, Alina O. Naumova, Vyacheslav A. Arlyapov, Olga A. Kamanina, Nadezhda M. Popova, Nikolay K. Zaitsev, Nikolay A. Yashtulov

**Affiliations:** 1 M. V. Lomonosov Institute of Fine Chemical Technologies, MIREA—Russian Technological University, Prosp. Vernadskogo 86, 119571 Moscow, Russia; 2 Federal State Unitary Enterprise Research and Technical Center of Radiation-Chemical Safety and Hygiene, Federal Medical-Biological Agency, 117105 Moscow, Russia; 3 Laboratory of Biologically Active Compounds and Biocomposites, Tula State University, Lenin Prosp. 92, 300012 Tula, Russia; 4 Federal State Budgetary Institution of Science Institute of Physical Chemistry and Electrochemistry of the Russian Academy of Sciences, Leninsky Prosp., 31 k. 4, 119071 Moscow, Russia; 5 Econics-Expert Ltd., Akademika Bakuleva St., 6, 117513 Moscow, Russia

**Keywords:** oxygen sensor, optical sensor, biosensor, microfluidics, lab-on-chip, modified nanodiamond, nanostructured surface, surface modification, adhesion control, fluorinated material, biofouling

## Abstract

In the last decade, there has been continuous competition between two methods for detecting the concentration of dissolved oxygen: amerometric (Clark electrode) and optical (quenching of the phosphorescence of the porphyrin metal complex). Each of them has obvious advantages and disadvantages. This competition is especially acute in the development of biosensors, however, an unbiased comparison is extremely difficult to achieve, since only a single detection method is used in each particular study. In this work, a microfluidic system with synchronous detection of the oxygen concentration by two methods was created for the purpose of direct comparison. The receptor element is represented by *Saccharomyces cerevisiae* yeast cells adsorbed on a composite material, previously developed by our scientific group. To our knowledge, this is the first work of this kind in which the comparison of the oxygen detection methods is carried out directly.

## 1. Introduction

The creation of sensors and biosensors for monitoring the environment is one of the most important priorities in science, because of the close connection between human health and socio-economic development. In this field, biosensors have been widely employed as cost-effective, fast, in situ, and real-time analytical techniques [1,2,3,4]. A biosensor usually consists of two main parts: a biological recognition element and a physical or chemical transducer [5]. The biological recognition part, in turn, can be one of three options: molecular, cellular, and tissue sensing components [6]. Various enzymes, DNA, antigens, antibodies, and biofilms are used as molecular recognizers. The significant advantage of these molecular-based biosensors is their high selectivity [7]. However, they have some disadvantages, such as the high cost of macromolecule isolation, limited detection capabilities, and a short lifetime of identifying molecules, which significantly limits the use of this type of bioreceptor [8].

In turn, sensors that are formed from cells or intact tissues have recently undergone rapid development in new methods of microfabrication and immobilization, and these very recent advances have provided these types of biosensors with unique advantages [9,10]. Whole-cell biosensors with prokaryotic or eukaryotic cells are typical examples of this type. The monitoring of various metabolic events in such systems is usually carried out through the analysis of the kinetics of respiration, which is a fundamental cellular process that responds to physical and chemical changes in the environment and is thus suitable for generating analytical signals [11]. Successful examples of such sensors include the detection of wastewater contamination [12], evaluation of petroleum hydrocarbon contamination [13], fermentation monitoring [14], and other technological processes [15].

The biochemical oxygen demand (BOD) parameter is used for monitoring the purity of an aqueous environment [16] and it represents the amount of dissolved oxygen required for the biochemical oxidation of organic substances contained in a sample at a certain temperature over a specific time period. Such an analysis takes five days according to the classical methodology [17], so considerable efforts are directed towards the development of methods for the rapid assessment of this characteristic using certain strains or communities of microorganisms [18,19,20].

Two types of transducers could be used to detect oxygen concentration as a measure of metabolic activity in whole-cell systems: electrochemical (Clark electrode) and optical (phosphorescence quenching of a tracer molecule) sensors [21]. It is important to note that they compete with each other [22], and so far both are used in the development of new analytical systems, since the choice of transducer is generally dependent on multiple factors, such as access to equipment and facilities, or substrate properties and materials of choice. Each of the methods has both advantages and disadvantages. In the case of the Clark electrode, the advantages are as follows: the method is well established, mechanically more robust than optical fiber sensors, and easily sterilized. The known disadvantages: consumption of O_2_ during measurement, the drift of readings over time and their dependence on hydrodynamic conditions, a more laborious maintenance procedure, and interfering action of a number of substances, such as H_2_S. Advantages of the optical method: it does not depend on the flow rate and salt background, it works well in electromagnetic fields, is stable after calibration, has a noninvasive readout, and planar sensors or nanosensors could be applied to imaging of O_2_. Disadvantages of optical measurement: it’s more expensive than electrodes, photobleaching of the dye can occur, it’s interfered with by chlorine but not by H_2_S, and has a brittle fiber tip (in the case of fiber sensors). Some works aim to identify a clear leader in this race, considering both the primary transducers themselves and biosensor systems as a whole. However, in the former case, it turns out that the uncertainties of the results for both analyzers are quite similar but the contributions of the uncertainty sources are different [23]. In the latter case, it is impossible to make a correct comparison, since the research conditions (strains, immobilization method, experimental conditions, target analyte, etc.) differ from article to article [21]. For this reason, one of the main goals of this work was to conduct a direct experimental comparison of two detection methods under identical conditions while working with the same receptor element in a synchronous mode. To our knowledge, this is the first work of this kind.

To achieve this goal, we decided to use microfluidic technology due to a number of advantages [24,25,26,27,28]. It allows for minimizing the volume of the analyzed sample, achieving good reproducibility of the hydrodynamic and temperature conditions of the experiment, which leads to a decrease in the dispersion of readings. Another advantage is the possibility of flexible modification and coupling of various stages of analysis in a compact device. These properties of the microfluidic system on the one hand make it an ideal tool for comparing two different methods, and on the other hand, largely eliminate the shortcomings of the electrochemical method associated with the influence of hydrodynamic conditions on the readings, and make the need for direct comparison of transduction methods obvious in order to identify the preferred way of organizing the measuring system. There are many cases of successful applications of this technology, for example, miniaturized electrochemical sensing platform or for measuring dissolved chemical oxygen demand (COD) in surface waters [29], antimicrobial susceptibility test (AST) using an optical oxygen sensor film for in-situ and real-time continuous measurement of extracellular dissolved oxygen [30], in-line analysis of organ-on-chip systems [31], and biomedical applications [32].

## 2. Materials and Methods

### 2.1. Reagents and Materials

The nutrient medium was prepared using D-glucose (Panreac, Barcelona, Spain), peptone (Condra, Spain), tryptone (Condra, Spain), and yeast extract (Helicon, Moscow, Russia).

Biosensor measurements were performed using a sodium-potassium phosphate buffer solution pH = 6.8 (33 mM KH_2_PO_4_ + 33 mM Na_2_HPO_4_, Dia-m, Moscow, Russia).

All other reagents and solvents used were chemically pure and produced by ChemMed (Moscow, Russia) and Sigma–Aldrich (Moscow, Russia).

### 2.2. Microfluidic Setup

**Figure 1:**
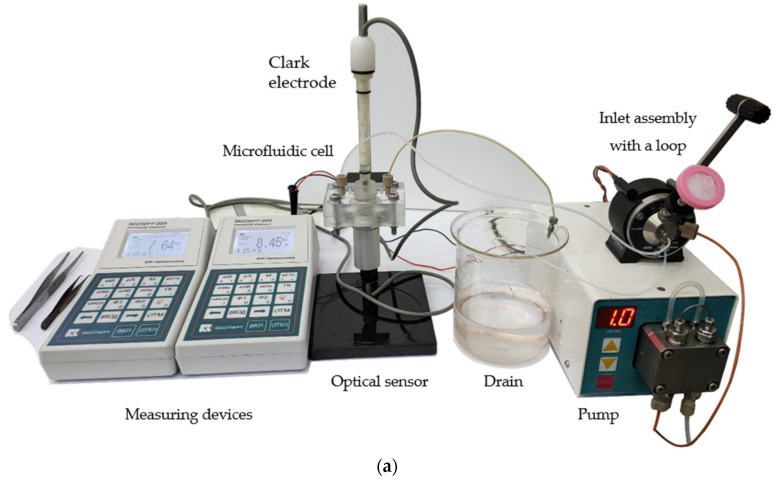

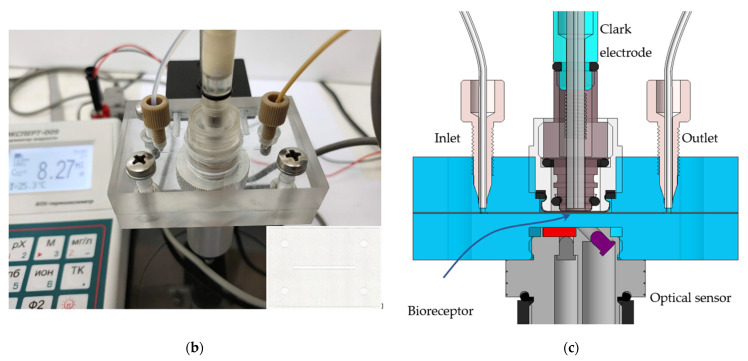
Experimental setup: (**a**)—overall view; (**b**)—the developed microfluidic cell with installed amperometric Clark electrode (**top**), optical oxygen sensor (**bottom**). A PET film with an eluent channel is shown in the inset; (**c**) schematic representation of a microfluidic cell in section, side view. The bioreceptor is placed in the center of the cell directly into the liquid flow, the amperometric (**top**) and optical (**bottom**) sensors are in close proximity, performing measurements in the same microvolume.

The experimental setup is shown in Figure 1. The eluent is pumped from the buffer tank using the software-driven HPLC-Pump K-120 (KNAUER Wissenschaftliche Geräte, Berlin, Germany), passes through the inlet assembly with a variable volume loop (Beckman Coulter, Brea, CA, USA), and then enters the specially designed microfluidic cell. Loops with a volume of 20, 50, 100, and 200 µL were used in the work.

The microfluidic cell (Figure 1b,c) consisted of two sheets of Plexiglas and a PET layer between them, fastened together by four screws. The PET film thickness was 100 µm. A channel for the flow of liquid 3 mm wide, as well as holes for fasteners, were made using a laser. The Clark electrode, model DKTP-02 (Econix-Expert, Moscow, Russia), was mounted in the center of the upper half of the cell. The optical oxygen sensor DKTP-03 (Econix-Expert, Moscow, Russia) was fixed in the center of the lower half of the cell. Both types of sensors were connected to the universal oxygen analyzer Expert-009 (Econix-Expert, Moscow, Russia) [33,34] whose readings were automatically transferred to a computer and synchronously collected by specially developed software. The inlet and outlet of the liquid flow was through HPLC tubes, which were fixed by standard 1/8 fittings with zero dead volume. This design of the cell made it possible to place two types of sensors as close as possible to each other so that measurements were carried out synchronously in the same microvolume in which the biosensor was placed.

### 2.3. Sensor Fabrication

A previously developed composite material was used as a substrate for the bioreceptor [35,36,37]. It consists of mesoporous SiO_2_ particles with an adsorbed indicator dye, which are evenly distributed in a gas-permeable fluorinated material. Pt (II) 5,10,15,20-tetrakis (2,3,4,5,6-pentafluorophenyl)-porphyrin (PtTFPP, Frontier Scientific, Logan, UT, USA) was adsorbed onto Merck silica gel 60 (Merck KGaA, Darmstadt, Germany) followed by distribution in fluorine-containing polymer, fluoroplastic 42 (F42, HaloPolymer, Moscow, Russia).

Formerly, we have shown that the introduction of modified detonation nanodiamond (DND) into the surface of a sensor material makes it possible to significantly vary its wettability, making it possible to control the adhesion of the biomaterial [38,39,40]. The described approach was also applied in this work. The methodology for the DND modification has been described in detail earlier [41]. UDA-GO-SP brand (Sinta, Minsk, Belarus) was used as starting material. Pristine DND was heated for 5 h in Ar-atmosphere with 3% of CCl_4_ at 400 °C. The obtained DND with acid chloride groups on the surface (DNDchl) were further heated in pure ammonia for 1 h at 1 atm and 300 °C. Partial substitution occurs under the described conditions, giving DND of a mixed composition (DNDamine + chl). The particle size distribution was measured using LS 13 320 XR Particle Size Analyzer (Beckman Coulter, Brea, CA, USA) to control possible particle aggregation during the treatment.

Preliminary studies have shown that it is DNDamine + chl in an amount of 4.6 × 10^−4^ g·cm^−2^ that makes it possible to achieve maximum surface biofouling [38,39], which is exactly necessary for the formation of a stable bioreceptor. The process of nanodiamond anchorage to the surface layer has been studied in detail earlier [40]. DNDs were placed in glycerol (12 g·dm^−3^) and the suspension was kept for 20 min in an ultrasonic bath. Then 30 μL was immediately applied to the samples. Annealing was carried out in a drying oven (SNOL-3,5,3,5,3,5/3,5-I2M, Thermix, Moscow, Russia) at the temperature of 160 °C in an argon atmosphere for 12 h.

The obtained material was tested in two types of experiments: (i) without biomaterial when sodium sulfite solution was introduced as a response simulator, (ii) with adsorbed yeast cells, and when glucose solutions of various concentrations or natural water samples were injected. In the latter case, the correlation between the analytical signal and the BOD_5_ parameter determined by the standard method was estimated.

### 2.4. Model Experiments

Model experiments were carried out with a sodium sulfite solution with a concentration of 0.5–5 g·dm^−3^ as a sample simulator to evaluate the response time and establish the optimal operating parameters for various operating modes of the created setup. The liquid flow rate was varied in the range from 0.5 to 2 ml·min^−1^ and the volume of the inlet loop was 20, 50, 100, and 200 µL.

### 2.5. Bioreceptor Formation

Pure cultures of the yeast *Saccharomyces cerevisiae*, known for rapidly accumulating biomass and easily forming biofilms, were used as a model biological object [42]. The cultivation in a liquid microbiological medium was carried out at 28 °C. The composition was as follows (g·dm^−3^): K_2_HPO_4_, 0.655; NH_4_Cl, 1.0; MgSO_4_ × 7H_2_O, 0.2; FeSO_4_ × 7H_2_O 0.01; CaCl_2_ 0.0075; Sucrose 50.0. Pre-sterilization of the medium was carried out for 40 min at 110 °C and 1.5 bar using an autoclave. Absorbance at 660 nm was used for biomass growth estimation (SF 2000 spectrophotometer, OKB Spectr, Saint-Petersburg, Russia). Goryaev chamber at 1200 magnification served for visual cell counting.

After the incubation time (4 days), the vial was opened, and the solution was centrifuged at 5000 rpm for 5 min using Hettich EBA 200 (Andreas Hettich GmbH & Co., Tuttlingen, Germany) to separate the medium (supernatant) from the cells. The resulting wet biomass was washed from the remnants of the medium with saline (0.9% NaCl solution) and centrifuged again. The procedure was performed several times. Thus, a concentrate of wet biomass in saline solution was obtained. 30 μL of suspension was applied to the sensor material. After drying at room temperature for 60 minutes, a finished bioreceptor was obtained. It was used immediately. At the end of the working day, the biosensor was disposed of.

### 2.6. Control of Biofilm Formation

The colorimetric MTT assay was used for the estimation of biofilm formation on the sensitive elements’ surfaces [43,44]. This method allows one to establish the viability of cells by assessing the cellular metabolic activity by the ability to reduce the yellow salt of 3-(4,5-dimethylthiazol-2-yl)-2,5-diphenyltetrazolium bromide (MTT). Samples were placed in 0.5 ml of a 0.1% MTT solution followed by incubation for 1 h at 29 °C. Then the liquid was drained, and stained samples were washed with water. An ethanol 96% was added and left for 45 minutes to extract the dye from biofilms. The optical density of the extract was determined at a wavelength of 590 nm using Expert-003 photometer (Econix-Expert, Moscow, Russia). The quality of the biofilm formation was judged by the color intensities of the obtained solutions.

### 2.7. Confocal Laser Scanning Microscopy (CLSM)

Confocal laser scanning microscopy (Leica SP5 microscope (Leica, Wetzlar, Germany)) made it possible to visualize the biofilm. Cells were stained with fluorescent dye SYTO^®^ 11 (S7573 ThermoFisher, Waltham, MA, USA) diluted 1:1000 in phosphate buffer. Lectin IV from wheat germ agglutinin (WGA) conjugated with fluorescent dye Alexa Fluor 488 (W11261 ThermoFisher, Waltham, MA, USA) was used for polysaccharide matrix staining [45]. The Nomarski contrast method allowed for detecting undyed particles. An argon laser with a wavelength of 488 nm (for detecting WGA fluorescence) and 594 nm (for detecting SYTO 11) was used. The resulting images were analyzed using the ImageJ software package with the BioFormats 5.8.2 plugin.

### 2.8. Determination of BOD by the Standard Dilution Method

The dilution method was used as a reference method for determining BOD_5_. The analysis was carried out in accordance with the procedure specified in [17]. Dissolved oxygen content was determined using an Expert-001-4.0.1 BOD thermooximeter (Econix-Expert, Moscow, Russia).

## 3. Results

### 3.1. Preliminary Experiments without Bioreceptor

**Figure 2:**
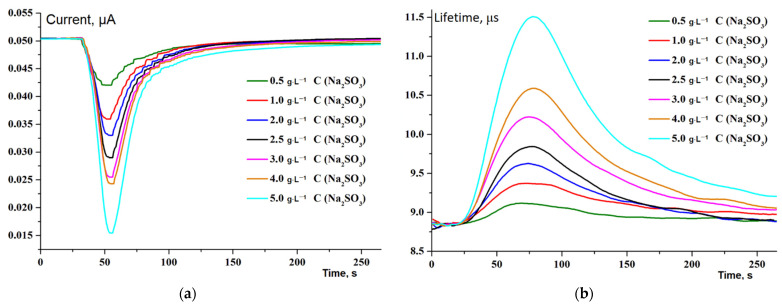
Dependences of the oxygen concentration in the analyzed microvolume on time from the moment the sample was injected into the flow for different concentrations: (**a**)—Clark electrode current; (**b**)—indicator dye excited state lifetime. Flow rate 1 mL·min^−1^, loop volume 0.2 ml.

Primary testing of the developed system was carried out without the use of a bioreceptor, using a sodium sulfite solution of various concentrations as a response simulator. The recording of the readings of both sensors began simultaneously with the injection of the sample into the flow. Examples of the observed dependencies (primary analytical signal vs. time) are shown in Figure 2. For the Clark electrode, the primary signal is the current in the electrochemical cell, which is directly proportional to the content of dissolved oxygen. In the case of the optical method, the lifetime of the excited state of the indicator dye is recorded, which is inversely related to the oxygen concentration by the well-known Stern-Volmer equation [46]:

τ_0_/τ = 1 + k_q_ τ_0_ [Q], (1)

where τ_0_ and τ are the lifetimes in the absence and the presence of the quencher Q, k_q_—is the bimolecular rate constant of the fluorescence quenching process due to a short-range interaction of species.

Figure 2 shows that both sensors respond to sample injection, but the reaction of the optical sensor starts a little earlier. The latter can be explained by the fact that the optical sensor is in direct contact with the analyzed solution, while the reaction in the Clark electrode occurs in an internal electrochemical cell separated from the liquid flow by a gas-permeable membrane that introduces a diffusion delay. This phenomenon may also be related to the higher detectable threshold of the Clark electrode, i.e., a visible response may require the reaction to reach a higher level in order to be detected.

**Figure 3:**
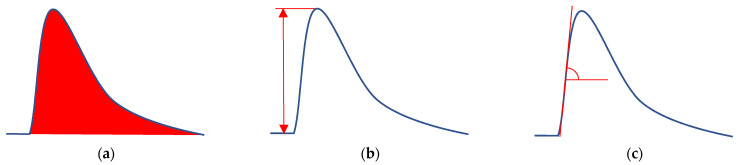
Methods for the recorded signal processing: (**a**)—calculation of the peak area; (**b**)—determination of the peak height; (**c**)—determination of the slope (the rate of change of the primary signal) after the sample enters the cell.

Registered dependencies can be processed in various ways to obtain an analytical signal. In this work, three methods were used: (i) calculation of the peak area, (ii) peak height determination, and (iii) slope calculation (rate of change of the primary signal) after the sample entered the microfluidic cell (Figure 3). In the latter case, a range of points was selected in the left half of the peak between two transition zones. The first one is due to the beginning of the sensor reaction to the sample entering the cell, and the second is due to the signal approaching its extremum. A linear trend line was superimposed on the selected points, and the slope was taken as an analytical signal. The criterion for discarding points falling into the transition zones was the correlation coefficient set for the linear trend in the form R^2^ ≥ 0.99.

**Figure 4:**
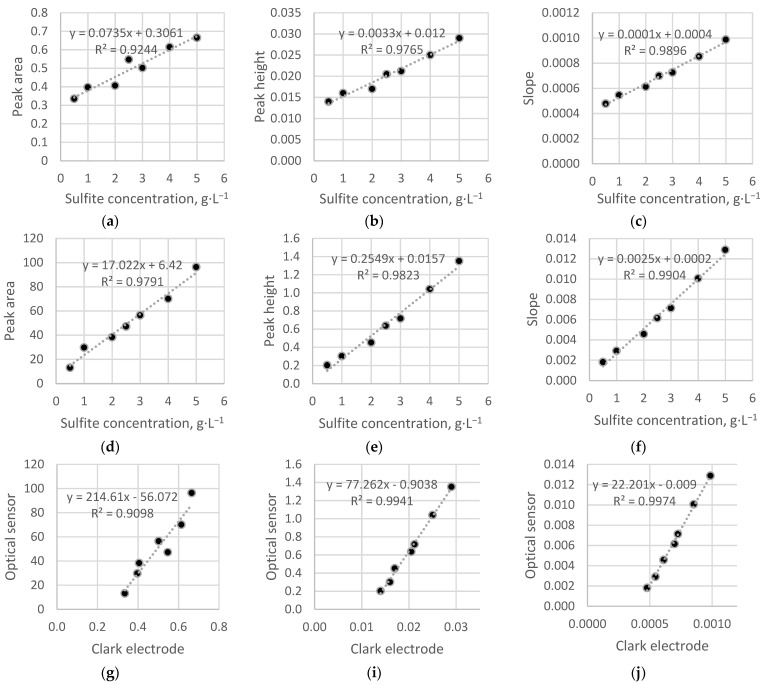
Calibration dependencies for different methods of the original data processing (calculated areas, heights, and slopes): (**a**–**c**)—Clark electrode; (**d**–**f**)—optical sensor. Readings of the optical sensor vs. Clark electrode: (**g**)—peak area; (**i**)—peak height; (**j**)—slope.

The results of the data processing for the preliminary experiment with sodium sulfite samples are shown in Figure 4. It can be seen that both types of sensors exhibit linear dependencies for all considered methods, but the best results were obtained when determining the slope. It is also worth noting that the dependencies for the Clark sensor, unlike the optical sensor, do not pass through the origin. This may be due to the principle of operation of this transducer, in particular, the presence of a background current and the dependence of the analytical signal on the hydrodynamic measurement conditions. The observed discrepancy between the calibration dependencies of the two types of sensors also indicates a lower threshold of determined concentrations of the optical sensor compared to the amperometric one. The limit of detection (LOD) for the electrochemical and optical sensors was 0.1 and 0.05 g·L^−1^, respectively. The limit of quantification (LOQ) was 0.5 and 0.2 g·L^−1^, respectively.

For further measurements, the slope factor processing was used, since it has the best linearity of response as well as the best correlation between the readings of the optical sensor and the Clark electrode. The determination of the peak area was abandoned to minimize the time of a single measurement. In addition, there was the worst correlation between the readings of the two sensors for this processing method.

### 3.2. Yeast Bioreceptor Properties

**Figure 5:**
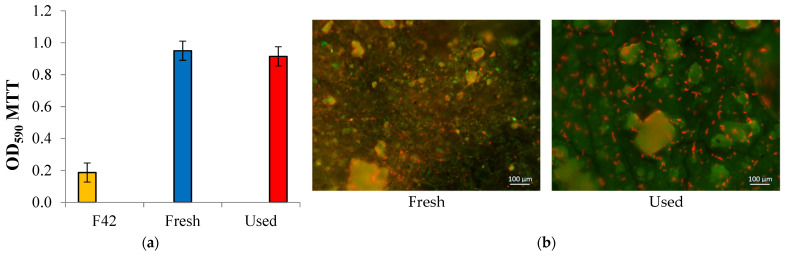
Properties of the created receptor element: (**a**)—respiratory MTT assay; (**b**)—Images of the surface (1200×) of a freshly prepared sensor with applied *Saccharomyces cerevisiae* yeast and a sample after a working day. Fluorescent staining: the cells are shown in red, and the polysaccharide biofilm matrix is indicated in green.

First, we wanted to make sure the cells are firmly attached to the surface of the substrate. For this, a freshly prepared receptor was compared with a receptor after a working day. A sensor substrate without biomaterial was used as a control. Figure 5a shows that the results of respiratory MTT assay differ little for the samples; the sensor during the working day practically did not lose its activity.

Photographs obtained by confocal laser scanning microscopy (Figure 5b) show that immediately after yeast application, there are quite a lot of cells on the surface (red staining, about 5% of the visible field), and their amount changes little after the sensor has been used for one working day. At the same time, the number of biofilms noticeably increases, which indicates favorable conditions for vital activity after the immobilization on the optical sensor surface. The optimal concentration of *Saccharomyces cerevisiae* yeast cells on the surface of the biosensor substrate was found to be 12.5 mg·cm^−2^. Such a bioreceptor demonstrated a stable and fast response to the substrate injection.

### 3.3. Application on Model Glucose Solutions

**Figure 6:**
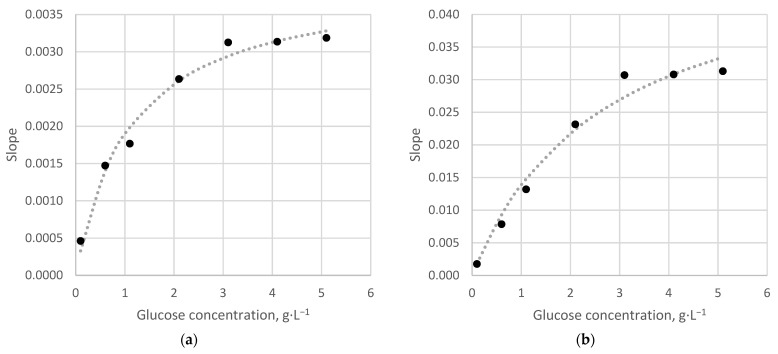
The response of the *Saccharomyces cerevisiae* bioreceptor on the concentration of glucose in the microfluidic cell: (**a**)—Clark electrode; (**b**)—optical oxygen sensor. Flow rate 1 mL·min^−1^, loop volume 0.2 mL.

The dependence of the response of the model bioreceptor on the concentration of glucose in the microfluidic cell was studied (Figure 6). Since a whole-cell receptor element, which is a catalytic-type bioreceptor, was used in this work, the resulting dependencies are described by the Michaelis–Menten equation:

(2) $$v=\frac{v_{\max}[S]}{K_{M}+[S]}$$

where *ν*—the rate of the enzymatic reaction; *ν*_max_—the sensitivity to the enzymatic reaction; *K_M_*—the apparent Michaelis constant; [*S*]—the initial concentration of the substrate.

**Figure 7:**
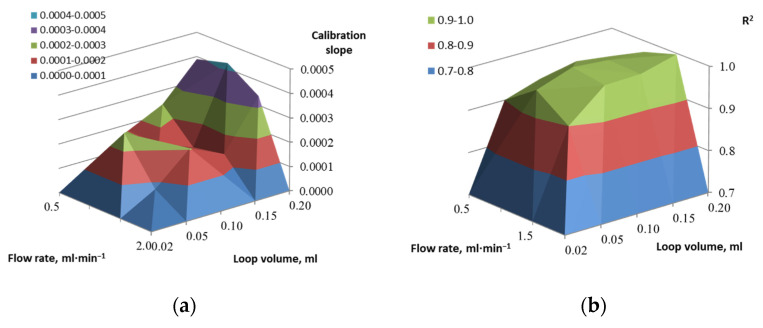

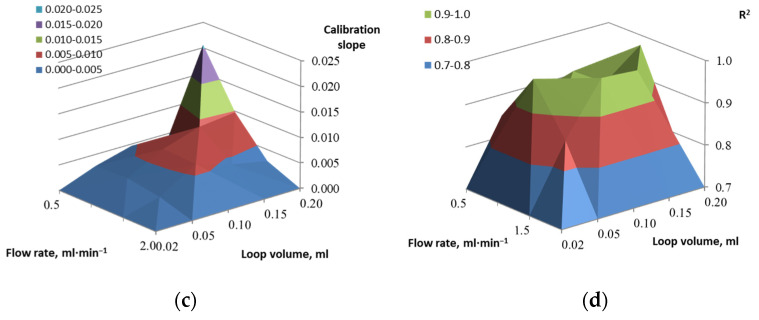
Influence of loop volume and flow rate on the system performance: (**a**)—calibration slope and (**b**)—coefficient of mixed correlation dependencies for Clark electrode; (**c**)—calibration slope and (**d**)—coefficient of mixed correlation dependencies for optical oxygen sensor.

The found value of the kinetic constant *K_M_* is 2.7 g·L^−1^, which is equal to such a concentration of the substrate at which the rate of the enzymatic reaction, is half of the maximum value. Therefore, the linear section of the calibration dependence is in the range of glucose concentrations from 0 to 2.0 g·dm^−3^, so further measurements were carried out in this range of substrate concentrations. The highest reproducibility of the analytical signal was determined by varying the volume of the loop (volume of the injected sample) from 0.02 to 0.20 mL and the flow rate from 0.50 to 2.00 mL·min^−1^. It was found that the optimal solution for both types of transducers was as follows: loop volume of 0.20 mL and a flow rate of 1 mL·min^−1^ (Figure 7). For these conditions, the dispersion of values is 18% for a glucose concentration of 0.5 g·dm^−3^ and less than 5% in the concentration range of 1–2 g·dm^−3^. The observed dependencies obtained for different types of primary data processing are close.

A comparison of optical and amperometric sensors shows that the optical sensor has better reproducibility and linearity of the calibration dependence. In addition, the slope of the calibration, built using the height of the peak, is 2 orders of magnitude, and in the case of calibration using the slope of the left side of the peak, it is an order of magnitude higher than the corresponding values for the Clark electrode.

### 3.4. BOD_5_ Estimation on Real Samples

**Figure 8:**
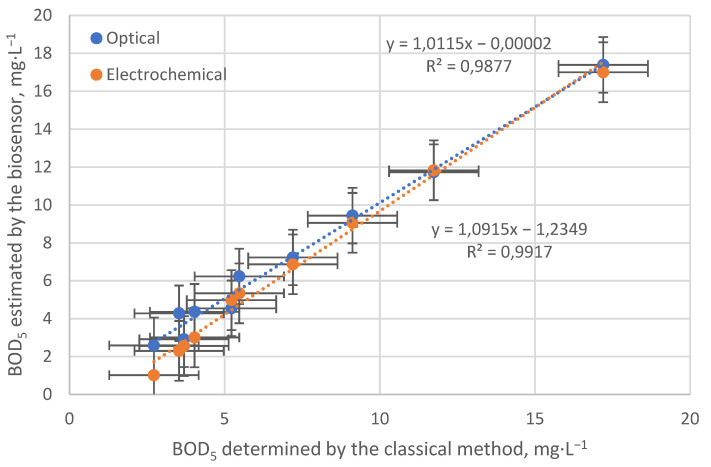
Dependence of the estimated BOD_5_ parameter on that determined by the classical method.

At the final stage of the study, a model bioreceptor based on Saccharomyces cerevisiae was also tested on a series of 10 real samples taken from several fish farm aquariums and wastewater. The main task was to identify the correlation between the analytical signal and the parameter BOD_5_, which characterizes the contamination of the sample with organic substances and is determined by a certified method [17]. The results are presented in Figure 8. It can be seen that, in general, the BOD_5_ values determined using different transducers are quite close to the values found by the classical method. However, the electrochemical sensor in the range of low concentrations exhibits an underestimation, and the overall slope of the correlation dependence significantly deviates from 1. This behavior of the Clark electrode makes it less attractive as a transducer in this kind of system, especially when operating in the low concentration range, when deviations are most noticeable. Moreover, it is important to consider other aspects of the two types of sensory modalities in addition to differences in analytical capabilities. The Clark electrode, although more cost-effective, is much more demanding on regular maintenance. A thin gas-permeable membrane is prone to mechanical damage, and the electrolyte may become contaminated and require replacement. In the case of the optical method, the measuring device itself does not come into contact with the analyzed medium, so no maintenance is required. The indicator dye is placed in a material of a small area and can be easily replaced after working out the resource. Exactly the same replacement will need to be carried out in the case of the electrochemical sensor since the cells are fixed on a special substrate, which requires periodic replacement.

## 4. Conclusions

The present work demonstrated for the first time a direct experimental comparison of two methods for measuring dissolved oxygen: electrochemical and optical. This goal was achieved through the integration of the sensors into the microfluidic system.

A yeast-based element was used as a receptor; the formation of biofilms was confirmed by a complex of methods. A practical example of synchronous measurement of the respiratory activity of model bioreceptor cells by two types of sensors clearly shows the advantage of the optical sensor in response time to changes in oxygen concentration, which is associated with the absence of an additional diffusion membrane layer. On the contrary, the indicator dye is located as close as possible to the bioreceptor element, allowing one to achieve better metrological characteristics during the optical measurement in the same process. The estimation of the BOD_5_ parameter using the created model bioreceptor made it possible to reveal the underestimation of the values measured by the electrochemical sensor in comparison with the certified method, while the optical sensor exhibited an almost 1:1 ratio. The known dependence of the readings of the electrochemical sensor on the liquid flow rate was not revealed under the conditions of the experiments, since all measurements were performed at a constant flow rate, which put both types of sensors on an equal footing when compared. However, a clear advantage of the optical measurement method was established as a result of the assessment.

The proposed methodological approach can be used for further comparative studies since the demonstrated advantage of the optical method in relation to *Saccharomyces cerevisiae* yeast does not mean that this will also be true for other biological objects. It is also important to note the good adhesive properties of the developed substrate used for cell immobilization. It can be used in the future to isolate the microbiome of a particular water body in order to assess its substrate specificity or resistance to toxic effects.

## Data Availability

Not applicable.
